# Antibody targeting tumor-derived soluble NKG2D ligand sMIC reprograms NK cell homeostatic survival and function and enhances melanoma response to PDL1 blockade therapy

**DOI:** 10.1186/s13045-020-00896-0

**Published:** 2020-06-09

**Authors:** Fahmin Basher, Payal Dhar, Xin Wang, Derek A. Wainwright, Bin Zhang, Jeffrey Sosman, Zhe Ji, Jennifer D. Wu

**Affiliations:** 1 Department of Microbiology and Immunology, Medical University of South Carolina, Charleston, SC 29425 USA; 2 Department of Urology, Feinberg School of Medicine, Northwestern University, Chicago, IL 60611 USA; 3 Driskill Graduate Program in Life Science, Feinberg School of Medicine, Northwestern University, Chicago, IL 60611 USA; 4 Department of Pharmacology, Feinberg School of Medicine, Northwestern University, Chicago, IL 60611 USA; 5 Department of Neurological Surgery, Feinberg School of Medicine, Northwestern University, Chicago, IL 60611 USA; 6 Department of Microbiology and Immunology, Feinberg School of Medicine, Northwestern University, Chicago, IL 60611 USA; 7 Division of Hematology and Oncology, Feinberg School of Medicine, Northwestern University, Chicago, IL 60611 USA; 8 Department of Biomedical Engineering, McCormick School of Engineering, Northwestern University, Evanston, IL 60628 USA; 9 Current address: Department of Medicine, Miller School of Medicine, University of Miami, Miami, FL USA

**Keywords:** NKG2D ligands, Soluble MHC I chain-related molecule (sMIC), PD1/PDL1 blockade, Anti-sMIC antibody, Melanoma

## Abstract

**Background:**

Melanoma patients who have detectable serum soluble NKG2D ligands either at the baseline or post-treatment of PD1/PDL1 blockade exhibit poor overall survival. Among families of soluble human NKG2D ligands, the soluble human MHC I chain-related molecule (sMIC) was found to be elevated in melanoma patients and mostly associated with poor response to PD1/PDL1 blockade therapy.

**Methods:**

In this study, we aim to investigate whether co-targeting tumor-released sMIC enhances the therapeutic outcome of PD1/PDL1 blockade therapy for melanoma. We implanted sMIC-expressing B16F10 melanoma tumors into syngeneic host and evaluated therapeutic efficacy of anti-sMIC antibody and anti-PDL1 antibody combination therapy in comparison with monotherapy. We analyzed associated effector mechanism. We also assessed sMIC/MIC prevalence in metastatic human melanoma tumors.

**Results:**

We found that the combination therapy of the anti-PDL1 antibody with an antibody targeting sMIC significantly improved animal survival as compared to monotherapies and that the effect of combination therapy depends significantly on NK cells. We show that combination therapy significantly increased IL-2Rα (CD25) on NK cells which sensitizes NK cells to low dose IL-2 for survival. We demonstrate that sMIC negatively reprograms gene expression related to NK cell homeostatic survival and proliferation and that antibody clearing sMIC reverses the effect of sMIC and reprograms NK cell for survival. We further show that sMIC/MIC is abundantly present in metastatic human melanoma tumors.

**Conclusions:**

Our findings provide a pre-clinical proof-of-concept and a new mechanistic understanding to underscore the significance of antibody targeting sMIC to improve therapeutic efficacy of anti-PD1/PDL1 antibody for MIC/sMIC^+^ metastatic melanoma patients.

## Introduction

Immunotherapy by blocking the axis of the immune checkpoint molecule programmed cell death protein (PD1) or its ligand PDL1 has presented remarkable survival benefit and thus became a frontline treatment for metastatic or unresectable melanoma [1, 2]. While the survival in advanced melanoma has improved substantially since FDA approved PD1/PDL1 blockade therapy, objective response rates approach only 50% at best with checkpoint inhibitors combination therapy [3]. Combination of anti-PD1 antibody and the antibody to the cytotoxic T cell lymphocyte-associated protein 4 (CTLA-4) slightly increased the response rate compared to anti-PD-1 monotherapy, however with significantly increased autoimmune toxicity [3]. Therefore, the need to increase the response rate with new combination therapy without increased toxicity is still imperative.

Identifying tumor-derived targetable factors that may impact patients’ response to PD1/PDL1 blockade would rationalize a potential combination therapy to improve the clinical outcome. In recent clinical studies, the presence of circulating soluble NKG2D ligands was shown to be negatively correlated with clinical outcome to anti-PD1/PDL1 response [4, 5]. In humans, there are two major family members of NKG2D ligands, the MHC I-chain-related molecules MICA and MICB and the viral HCMV UL16-binding proteins ULBP1-6 family. NKG2D ligands are rarely expressed by normal tissues unless under stress insults, such as infection [6], but induced in most tumor cells in part through activation of DNA damage response pathway or oxidative stress [7–9]. Although the MICA and MICB family molecules are better characterized and more prevalently expressed than the ULBP family proteins, these ligands often co-exist in one tumor type, presumably through host-viral co-evolutionary processes [10]. While levels of the NKG2D ligand MIC have been correlated with survival benefits in the early stages of several cancers, the opposite has been demonstrated with more invasive tumors [11–14]. In most invasive human tumors, NKG2D ligands also exist as a soluble form through proteolytic shedding or exosome secretion [15]. Soluble human NKG2D ligands have been shown to subvert antitumor immunity through multiple mechanisms, including but not limited to, perturbing NK cell homeostatic maintenance and function, impairing CD8 T cell function by destabilizing CD3ζ [16], and expanding myeloid-derived suppressive cells (MDSCs) in the tumor microenvironment [17]. These mechanistic understandings along with the reported clinical observations prompt us to test the hypothesis that co-targeting tumor-derived soluble NKG2D ligands would enhance melanoma tumor response to PD/PDL1 blockade therapy.

We have previously described that clearance of tumor-derived soluble NKG2D ligands, sMIC, with a monoclonal antibody (mAb) B10G5 restores NK cell homeostatic renewal, enhances NK cell and antigen-specific CD8 T cell function, immobilizes NK and CD8 T cell to the tumors, and re-modulates tumor microenvironment by eliminating MDSCs and tumor-associated macrophages [17, 18]. In this study, we demonstrate that antibody targeting sMIC increases the IL-2 sensing receptor IL-2Rα on NK cells, reprograms NK cell homeostatic maintenance, and enhances the therapeutic response of melanoma tumors to PD1/PDL1 blockade therapy. Our current study provides a new mechanistic understanding to accentuate the significance of co-targeting tumor-derived soluble NKG2D ligand, sMIC, to enhance the therapeutic efficacy of PD1/PDL1 checkpoint blockade therapy for melanoma patients.

## Materials and methods

### Mice and cell lines

Mice were bred and housed under specific pathogen-free conditions in the animal facility of the Medical University of South Carolina and Northwestern University in accordance with institutional guidelines with approved IACUC protocols. All mice used in this study were male rPB-MICB mice on the B6 background as previously described and thereafter defined as MICB/B6 mice [19]. sMIC-expressing B16F10-sMICB cell line was developed by transduction of B16F10 cells (ATCC) with an IRES-GFP retroviral vector containing the construct for recombinant soluble MICB, as described previously [20]. sMIC^+^ B16F10 cells were selected by puromycin and further by flow cytometry sorting for GFP-positive cells.

### Antibodies, peptides, and tetramers

InVivoMAb anti-mouse PDL1 (clone 10F.9G2) was purchased from BioXCell. Generation of the anti-MIC mAb B10G5 was previously described [19]. B10G5 is a mouse IgG1 isotype, recognizing both MICA and MICB. B10G5 binds to free sMIC but does not block the interaction of sMIC with the receptor NKG2D [18]. B10G5 was produced and purified from the hybridoma culture by BioXCell (West Lebanon, NH). In vivo NK cell-depleting anti-NK1.1 (clone PK136) and CD8 T cell-depleting antibody anti-CD8α (clone 2.43) were purchased from BioXcell. Peptide gp100_25-33_ (KVPRNQDWL) was synthesized by GenScript. H-2D^b^/ gp100_25-33_ tetramer was produced by NIH Tetramer Core Facility at Emory University.

### Tumor inoculation and in vivo experiments

For subcutaneous study, B16F10-sMICB cells were implanted subcutaneously into the right flank of cohorts of syngeneic MICB/B6 male mice (4 × 10^5^ cells/mouse) at ages 8–10 weeks old. When tumor volume reached approximately 75–100 mm^3^, animals were randomized into four therapy groups (*n* = 5 to 7 per group): (1) control mouse IgG (3.0 mg/kg BW), (2) anti-MIC mAb B10G5 (3.0 mg/kg BW), (3) anti-PDL1 mAb (3.0 mg/kg BW), and (4) B10G5 and anti-PDL1 mAb. All antibodies were given via I.P. injection every 3 days. For survival studies, tumor volume of 1800 mm^3^ was defined as survival endpoint. For mechanistic studies, animals were euthanized after 9 days of treatment. After euthanization, the spleens and two inguinal draining lymph nodes (dLN) and tumors were harvested. Partial of the tumors were formalin fixed, paraffin embedded, and sectioned for histology and immunohistochemistry staining (IHC). The remaining tumors were used for single-cell suspension preparation by the method of mincing, mechanically processing, and passing through a 70-μm filter. Single-cell suspension of splenocytes, dLN, and tumors was used for ex vivo stimulation and flow cytometry analyses.

For lung metastasis, B16F10-sMICB cells were injected into the lateral tail vein of syngeneic B6/MICB male mice (2 × 10^5^ cells/mouse) at ages 8–10 weeks old. At day 10 post-tumor inoculation at which time point tumor nodules were visible on the surface of the lung by random examination of three animals, mice were randomized into four therapy groups (*n* = 5 per group): (1) control mouse IgG (3.0 mg/kg BW), (2) anti-MIC mAb B10G5 (3.0 mg/kg BW), (3) anti-PDL1 mAb (3.0 mg/kg BW), and (4) B10G5 and anti-PDL1 mAb. All antibodies were given via I.P. injection every 3 days. Animals were euthanized at day 21 following tumor inoculation. Spleens, inguinal draining lymph nodes, and lungs were harvested for analyses.

### Ex vivo cytokine re-stimulation assay

For general re-stimulation, single-cell suspensions of splenocytes and draining lymph nodes were stimulated at 37°C for 6 h with 50 ng/ml phorbol myristate acetate (PMA) and 500 ng/ml ionomycin. To assess melanoma antigen-specific T cell function, single-cell suspension of bulked splenocytes or tumor-draining lymph nodes was stimulated with 1 μg/ml of melanoma antigen gp100_25-33_ peptides overnight. IFNγ production was assayed by intracellular staining with BD IFNγ staining Kits following the manufacturer’s instruction.

### Flow cytometry analysis

Single-cell suspensions were incubated on ice for 30 min with a combination of antibodies specific to cell surface markers for identification of lymphocyte subsets. These antibodies are anti-NK1.1 (clone PK136), anti-CD3 (clone 145–2C11), anti-CD8α (clone 53–6.7), anti-NKG2D (clone CX5), anti-CD44 (clone IM7), anti-CD25 (clone PC61), anti-Gr1 (clone RB6-8C5), and anti-CD11b (clone ICRF44). All antibodies used for flow cytometry analyses were purchased from Biolegend (San Diego, CA, USA). Tetramer staining was performed with 2 μg/ml of PE-labeled H-2D^b^/ gp100_25-33_ tetramer at 37 °C for 20 min and followed by surface marker staining. For intracellular staining, cells were stained with surface markers followed by fixation and permeabilization with BD Perm/Fix kits and antibodies specific to intracellular molecules. Cells were analyzed using the BD Fortessa. Data were analyzed using the FlowJo software (Tree Star).

### Histological and immunohistochemistry staining

Five micrometers of formalin-fixed paraffin-embedded sections were stained with H&E for pathological evaluation and used for immunohistochemistry (IHC) staining. Mouse tumor sections were also stained with the following: (a) anti-NKp46/NCR1 (rabbit IgG; 1:200; Abcam); (b) anti-CD8 (BD biosciences); (c) anti-arginase 1 (rabbit IgG; 1:200; Santa Cruz Biotechnology); (d) anti-CD31 (rabbit IgG; 1:100; Abcam); and (e) anti-Ki67 (rabbit IgG, Abcam, 1 μg/ml). Human tissue microarray (TMA) sections containing 62 cases of malignant melanoma, 21 metastatic malignant melanoma, and other control tissues were purchase from US Biomax (Cat. ME1004g) and were stained with the mouse monoclonal anti-MIC antibody D4H3 (Supplement Material and Methods). Sections were deparaffinized and incubated for 10 min in 10 mM citrate buffer (pH 6.0) at 95 °C for antigen retrieval. Endogenous peroxidase activity was quenched with 3% hydrogen peroxide. After quenching endogenous peroxidase activity and blocking nonspecific binding, slides were incubated with specific primary antibody overnight at 4 °C followed by subsequent incubation with the appropriate biotinylated secondary antibody: goat anti-rabbit IgG or goat anti-mouse (Vector) at a 1:1000 dilution for 20 min at 37 °C. Immunoreactive antigens were detected using the Vectastain Elite ABC Immunoperoxidase Kit and DAB. All slides were counterstained with hematoxylin (Vector) and mounted with Permount (Fisher Scientific).

### RNAseq and data analyses

Single-cell suspension from the spleens from SCID mice was prepared as described [20]. After removal of adherent cells for 2 h in complete media, splenocytes were cultured in media containing 1000 U/ml IL-2 for 3 days. NK cells were negatively selected with EasySep™ mouse NK isolation kits (StemCell Technologies). A 99% purity was obtained. Purified NK cells were cultured with purified recombinant sMIC(B)-His (Sino Biologicals) or sMIC(B)-Fc (R&D) for 12 h, with or without the presence of the anti-sMIC mAb B10G5. Total RNA was prepared with RNeasy kit (Qiagen). RNAseq library were constructed with Illumina TruSeq Stranded mRNA Library Prep Kit. Twenty to 32 million of RNAseq reads were obtained for each sample using single-end 50 bp sequencing. Trim Galore (https://www.bioinformatics.babraham.ac.uk/projects/trim_galore/) was used to validate the quality of the reads and to remove ones with low quality by default parameters. STAR program was used to align the reads against the mouse reference genome mm10 with the transcriptome annotation GTF file from ENSEMBL (GRCm38.82) via the default parameters [21]. FeatureCounts was used to calculate gene expression, represented as transcript per million (TPM) values [22]. Only genes showing TPM value greater than 4 in at least one sample were included in the downstream differential gene expression analyses. DAVID online tool (http://david.abcc.ncifcrf.gov/) was used for the gene enrichment analysis. Genes showing consistent differential expression (> 2 fold in two biological replicates) upon sMIC treatments as compared to the control sample were selected for the Gene Ontology (GO) analysis and for the heatmap.

### Quantitative RT-PCR

Total RNA was prepared as described above. Complementary DNA (cDNA) was synthesized using the SuperScript II kit (Invitrogen). A volume of 1 μl of cDNA was mixed with Power SYBR Green qPCR SuperMix (Bio-Rad, USA), and specific primer sets were added to a final concentration of 400 nM in 20 μl of reaction mixture. The reaction was performed on a Bio-Rad CFX96 Touch Real-Time PCR Detection System. Data were analyzed using CFX Maestro Software (BioRad). Each sample was assayed in triplicates. Target mRNA levels were normalized against mouse GAPDH. Gene expression level in control NK cells was used as a reference for calculating expression fold changes. The primers used are listed in Supplement Table S1.

### Statistics

All statistical data were expressed as mean ± SEM. Difference between means of populations was compared by standard Student’s *t* test using one-way ANOVA. Survival was determined via Kaplan-Meier analysis with a comparison of curves using the Mantel-Haenszel log-rank test. A *P* value of 0.05 or less was considered significant. GraphPad Prism software was used for all analyses.

## Results

### Targeting sMIC in combination with PD1 blockade cooperatively increased survival in mice bearing sMIC-positive melanoma tumors

**Fig. 1:**
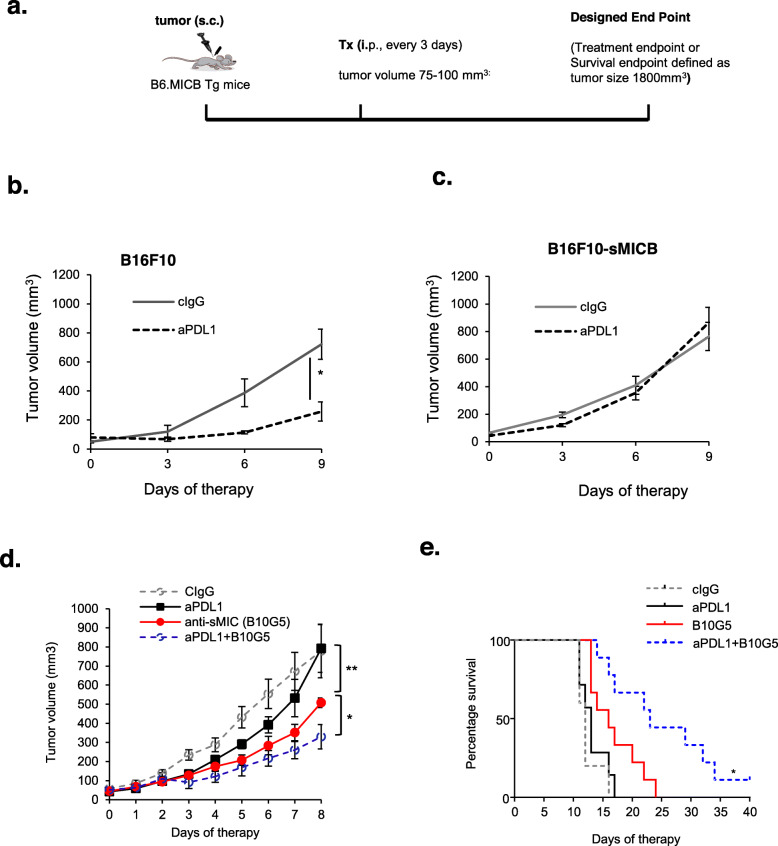
sMIC compromises tumor response to anti-PDL1 mAb therapy and that antibody targeting sMIC generates cooperative therapeutic effect with anti-PDL1 mAb. **a** Depict of the therapy. B16F10 or sMICB-expressing B16F10-sMICB cells (4 × 10^5^ cells/mouse) were *s.c.* injected into syngeneic MICB/B6 host. Therapy initiated when tumors reached a volume of 75–100 mm^3^. Antibody (3 mg/kg) was given *i.p.* twice weekly till designated study endpoint as specified in the “Materials and methods” section. **b**, **c** Therapeutic response of B16F10 (**b**) and B16F10-sMICB (**c**) tumors to anti-PDL1 therapy. Data showed a compromised response of B16F10-sMICB tumors to anti-PDL1 therapy. **d** B16F10-sMICB tumor growth curve in response to sMIC-targeting antibodyB10G5 and anti-PDL1 antibody single-agent therapy and combination therapy. **e** Kaplan-Meier survival curve showing that B10G5 and anti-PDL1 combination therapy significantly prolonged survival of mice bearing B16F10-sMICB tumors. Note that tumor volume of 1800 mm^3^ was designated as survival endpoint. *N* = 5–7 per group, *p < 0.05; **p < 0.01

Melanoma patients who have high levels of circulating sMIC responded poorly to PD1/PDL1 blockade therapy [4, 5]. To test whether our animal model recapitulates the biology in melanoma patients, we utilized a transplantable B16F10-sMICB syngeneic tumor model as previously described [19, 20]. Briefly, since mice do not express homologs of the human ligand MICA/B, we utilized MICB/B6 male transgenic mice as a syngeneic host to prevent potential unwanted immunogenicity against human sMICB overexpressed by mouse tumors (Fig. 1a). The MICB/B6 transgenic mice have an inducible MICB expression under the control of the male hormone-sensitive promoter probasin and tolerant to sMICB-expressing tumors [19]. As shown in Fig. 1b, c, anti-PDL1 antibody treatment significantly inhibited B16F10 tumor growth but had nominal impact on the growth of B16F10-sMICB tumors. These data suggest that the B16F10-sMICB syngeneic tumor model recapitulates the biology of sMIC in melanoma patients in the context of response to PD1/PDL1 blockade therapy.

To test the hypothesis that targeting the soluble NKG2D ligand MIC enhances therapeutic efficacy of PD1 blockade, we randomized a cohort of B16F10-sMICB tumor-bearing mice into four therapeutic groups (*n* = 8–10 per group) with control IgGs (cIgG) in vehicle PBS, single-agent sMICB-targeting mAb B10G5 or anti-PDL1 mAb, or an antibody cocktail of B10G5 and anti-PDL1 mAb. Consistent with our previous observation [18], monotherapy with mAb B10G5 significantly inhibited tumor growth and extended survival as compared to control or anti-PDL1 monotherapy (Fig. 1d, e). Remarkably, the combination of anti-PDL1 and B10G5 resulted in a further significant inhibition of tumor growth and improved survival as compared to monotherapy of B10G5, suggesting a cooperative therapeutic effect of targeting sMIC and PD1/PDL1 blockade. These results were further corroborated in a syngeneic RM9-sMICB prostate tumor model (Supplement Figure S1).

### Combination therapy remodels tumor microenvironment to suppress tumor growth

**Fig. 2:**
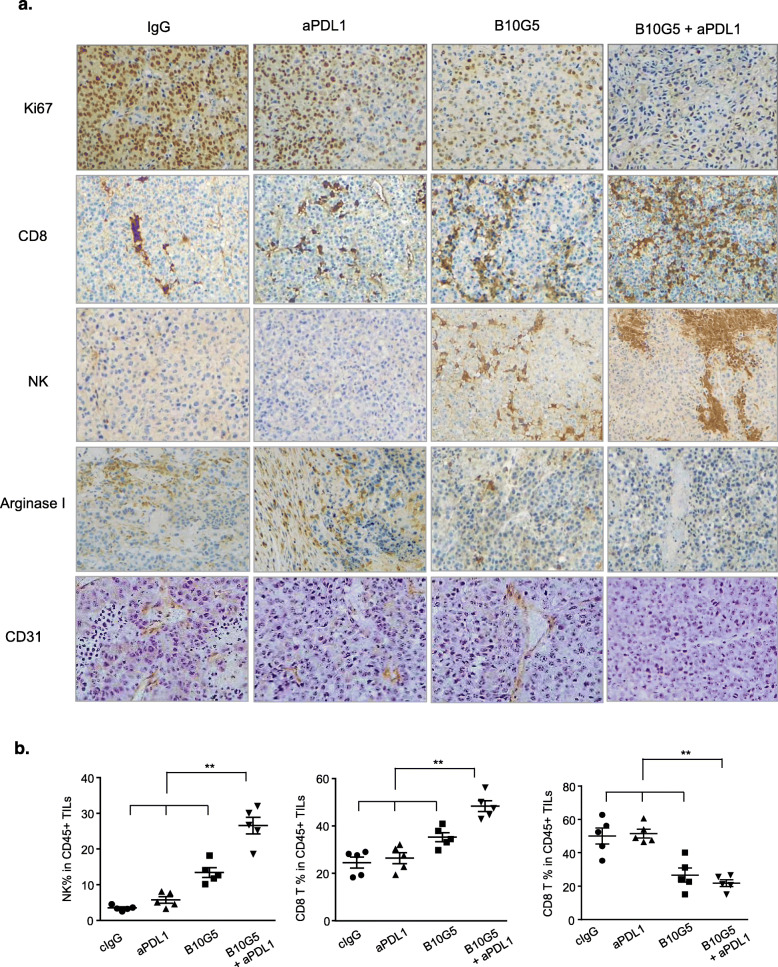
Combined therapy of B10G5 targeting sMIC and anti-PDL1 results in reduced tumor proliferation and a more immune primed tumor microenvironment with decreased neovascularization. **a** Representative micrographs of immunohistochemistry staining (IHC) of subcutaneous B16-sMICB tumors demonstrating that combined therapy resulted in reduced tumor cell proliferation as shown by Ki67 staining, increased NK and CD8 T cell, and decreased arginase 1^+^ cells in tumors. Combined therapy also decreased neovascularization within the tumor shown by CD31 staining. **b** Quantitation of NK cell, CD8 T cell, and the major arginase I producer MDSC in representative tumors by flow cytometry analyses. Data obtained at day 8 following treatment initiation in an experiment designed to understand therapeutic mechanisms as detailed in the text. *p < 0.05 as compared to the control group. **p < 0.05, combination therapy as compared to monotherapy

We assessed treatment on tumor proliferation by immunohistochemistry (IHC) staining of tumor sections with ki67. While therapy with anti-PDL1 mAb did not significantly inhibit tumor proliferation, treatment with the sMIC-targeting mAb B10G5 evidently reduced tumor proliferation as compared to control cIgG treatment (Fig. 2a). The combination therapy of anti-PDL1 mAb and B10G5 resulted in a remarkable inhibition of tumor cell proliferation as compared to therapy with single-agent or cIgG (Fig. 2a).

Anti-PDL1 antibody single-agent therapy did not significantly impact intra-tumoral lymphocyte infiltration although a trend of increased density of CD8 T cells was observed (Fig. 2a, b). B10G5 single-agent noticeably increased the density of CD8 T and NK cells in tumors. Therapy with the combination of B10G5 and anti-PDL1 mAb remarkably enriched CD8 and NK cells in tumors as compared single-agent therapy (Fig. 2a, b). In some cases, tumors were encased by NK and CD8 T cells (Fig. 2a). Arginase I^+^ cells in tumor infiltrates are considered a hallmark for tumor-promoting macrophages and myeloid-derived suppressor cells [23–25]. Control IgG-treated tumors were infiltrated with a high density of arginase I^+^ cells (Fig. 2a). Single-agent therapy with B10G5, but not anti-PDL1 mAb, reduced the infiltration of arginase I^+^ cells at a significant level (Fig. 2a). Arginase I^+^ cells were rarely found in the tumors of mice that received combination therapy (Fig. 2a). Consistently, the number of MDSCs, the major producer of arginase I, was significantly reduced in tumors with combination therapy as compared to single-agent therapy (Fig. 2b). In accordance with this observation, tumor vasculature as represented by CD31 IHC staining was evidently the combination therapy (Fig. 2a). These data demonstrate that the anti-PDL1 mAb and B10G5 act cooperatively to modulate tumor microenvironment to be more active immune-primed for anti-tumor responses.

### Combination therapy cooperatively augmented tumor CD8 T cell intrinsic and melanoma anti-specific responses

**Fig. 3:**
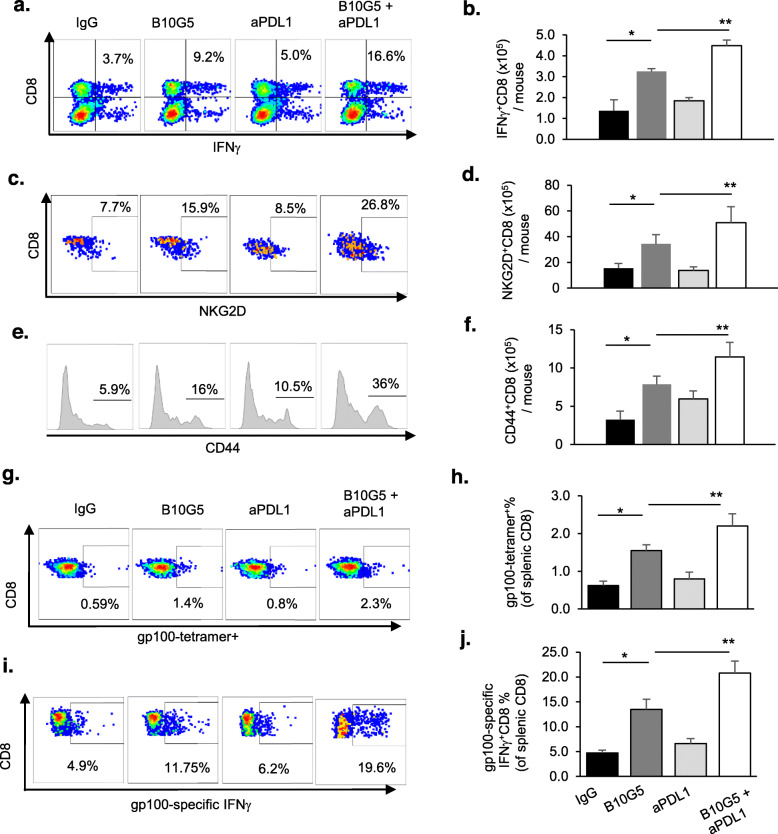
Combined therapy with B10G5 and anti-PDL1 increases intrinsic functional potential and response to antigen-specific stimulations. B16F10-sMICB cells (4 × 10^5^ cells/mouse) were *s.c*. injected into syngeneic MICB/B6 host. When tumors reached a volume of 75–100 mm^3^, animals received *i.p* injection of 3 mg/kg of respective antibody every 3 days. After three injections (day 9 of therapy), animals were euthanized. Tissues were harvested for therapy-associated mechanistic studies. **a**, **b** Combined therapy significantly increases IFNγ-producing splenic CD8 T cells as assessed by ex vivo PMA/ionomycin stimulation. **c**, **d** Combined therapy significantly increased the number of splenic NKG2D^+^ CD8 T cells. **e**, **f** Combined therapy significantly increased the population of CD44^+^CD8 T cells. Data obtained at day 14 following treatment initiation. **g**, **i** Representative flow cytometry dot-plots demonstrating that combined therapy significantly increases gp100-tetramer^+^ CD8 T cell and increased IFNγ expression with gp100 peptide stimulation. Single-cell suspension of splenocytes was stained with melanoma antigen gp100-specific tetramer or evaluated for CD8 T cell IFNγ expression after stimulation with gp100 peptide overnight. **h**, **j** Summary data from the experiments presented in **g** and **i** respectively. *p < 0.05 as compared to the control group. **p < 0.05, combination therapy as compared to monotherapy

To understand the underlying mechanisms of the combined therapeutic effect, we first evaluated how each therapy modulated the anti-tumor potential of CD8 T cells. We first assessed the impact of therapy on intrinsic CD8 T cell functional potential by IFNγ production in response to ex vivo re-stimulation with PMA/ionomycin. Single-agent therapy with B10G5 significantly increased the total number of splenic IFNγ^+^ CD8 T cells, whereas anti-PDL1 therapy alone did not present such a significant impact (Fig. 3a, b**)**. Combined therapy resulted in further significantly increased responsiveness of splenic CD8 T cells as compared to each monotherapy (Fig. 3a, b).

One of the significant immune-suppressive effects of soluble NKG2D ligands is systemic downregulation of NKG2D expression on CD8 T cells to diminish the co-stimulatory pathway of CD8 T cells [26]. B10G5 single-agent therapy significantly increased the percentage of NKG2D^+^ CD8 T cells in the spleen. Although anti-PDL1 mAb single agent therapy did not result in a significant impact on NKG2D expression on CD8 T cells, the combination of B10G5 and anti-PDL1 mAb significantly increased the percentage of NKG2D^+^ CD8 T cells compared to B10G5 therapy (Fig. 3c, d). NKG2D is constitutively expressed by all human CD8 T cells; however, it is only expressed by activated mouse CD8 T cells [27]. Hence, these observations indicate an increased activation of CD8 T cells in response to therapies. Consistently, effector memory CD8 T cells represented by CD44^+^ CD8 T cells were significantly enriched with the combination therapy (Fig. 3e, f).

We further investigated antigen-specific CD8 T cell responses with monotherapy and combination therapy. Melanoma antigen gp100-specific CD8 T cell populations, as represented by H-2D^b^-restricted gp100-tetramer^+^ population, were significantly enriched in tumors with B10G5, but not with anti-PDL1, single-agent therapy (Fig. 3g, h). Combination therapy of anti-PDL1 and B10G5 significantly enriched gp100-tetramer^+^ CD8 T cells as compared to B10G5 therapy single-agent therapy (Fig. 3g, h). Moreover, the gp100-tetramer^+^ CD8 T cells was significantly more responsive to ex vivo gp100 peptide re-stimulation with B10G5 therapy than control IgG or anti-PDL1 therapy, as measured by intracellular IFNγ staining (Fig. 3i, j). Combination therapy of B10G5 and anti-PDL1 further enhanced the responsiveness of gp100-tetramer^+^ CD8 T cells to gp100 peptide re-stimulation (Fig. 3i, j). Together, these data demonstrate that antibody targeting sMIC in combination with PD-1/PDL1 blockade cooperatively increase CD8 T cell intrinsic ability to respond to stimulation and augments antigen-specific CD8 T cells in the tumors.

### Co-targeting sMIC during anti-PDL1 therapy increases CD25 expression on NK cells and augments NK cell homeostatic maintenance

**Fig. 4:**
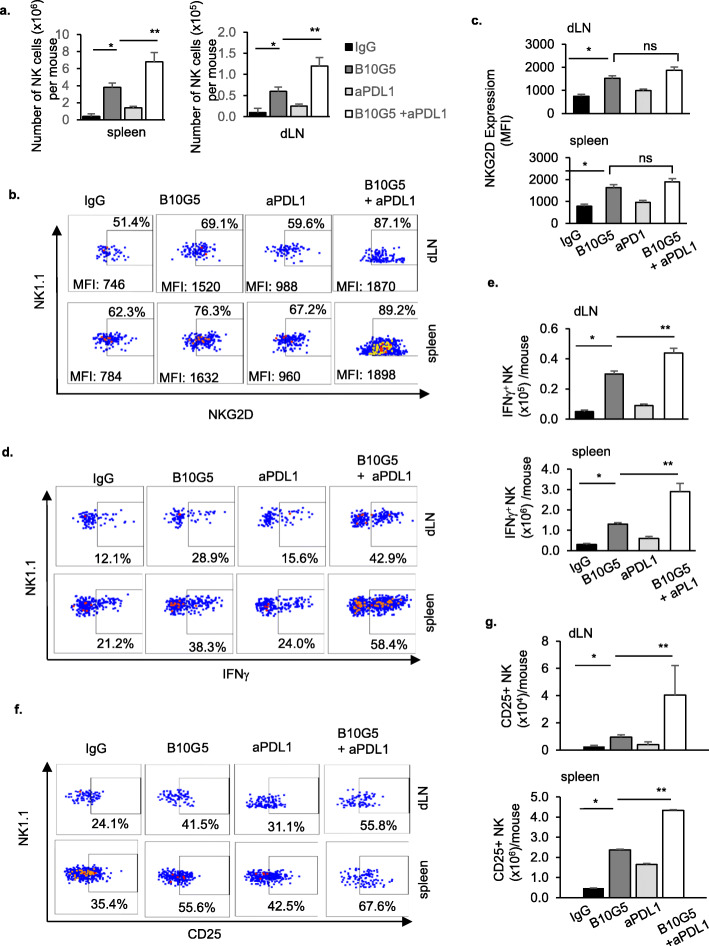
Combined therapy cooperatively restores NK cell homeostatic function and increase IL-2Rα expression on NK cells. Data associated experimental details are described in Fig. 3 legend. **a** Total number of NK cells harvested from spleen and two inguinal draining lymph nodes per mouse. **b**, **c** Monotherapy with B10G5 and/or anti-PDL1 increases intensity of NKG2D expression on NK cells in the draining LN of mice bearing B16-sMICB tumors, while combined therapy further increases NKG2D expression. **d**, **e** Combined therapy increases IFNγ production after ex vivo PMA/ionomycin stimulation of NK cells from the draining LN of mice bearing B16-sMICB tumors. **f**, **g** Combined therapy significantly increases numbers of NK cells in tumor-draining lymph nodes expressing CD25. *p < 0.05 as compared to the control group. **p < 0.05, combination therapy as compared to monotherapy

Soluble NKG2D ligand sMIC negatively affects the maintenance of peripheral and tumor-infiltrating NK cells and downregulates NKG2D surface expression [19]. Targeting sMIC with mAb B10G5 has been shown to overcome the disruption and to restore NK cell homeostatic renewal ability [18]. Given that PD1 blockade therapy was shown to restore NK cell anti-tumor activity [28], we thus sought to determine the impact of the combination therapy on NK cells. As shown in Fig. 4a, combination therapy significantly increased NK cell numbers in the spleen and dLN in addition to the significant increase in tumors as presented in Fig. 1 suggesting an enhanced NK cell homeostatic maintenance. NKG2D expression was significantly restored or increased with B10G5 therapy, although only a trend of increase with anti-PDL1 therapy. Combination therapy significantly restored surface NKG2D expression as compared to single agent therapies, although no significant additive or cooperative effect (Fig. 4b, c). NK cell intrinsic ability in response to ex vivo stimulation, such as PMA/I, was significantly augmented by B10G5 therapy and further by combination therapy (Fig. 4d, e).

Interestingly, combination therapy induced more NK cells to express the IL-2Rα, CD25 (Fig. 4f, g), which most likely couples with the common γ-chain family receptors (IL-2/IL-15Rβ and γ_c/_IL-2Rγ) to form the high affinity IL-2 receptor to enable NK cells to utilize low-dose IL-2 sourced from activated T cells for survival and proliferate [29, 30]. To support this hypothesis, we measured serum levels of IL-2 before therapy and at day 8 of therapy. While only neglectable levels of IL-2 were detected in the serum of all animals before therapy, a significant amount of IL-2 was detected in the serum of animals receiving therapy of B10G5 or B10G5 in combination with anti-PDL1 (Supplement Figure S2). These data suggest that B10G5 co-targeting sMIC likely restores NK homeostatic maintenance and thus NK cell-mediated anti-tumor immunity through regulating the availability of IL-2 and sensitivity of IL-2 signaling on NK cells, as compared to anti-PDL1 single agent therapy.

We further addressed the significance of NK cells and CD8 T cells in mediating the cooperative therapeutic effect of targeting sMIC and anti-PDL1 by depleting NK or CD8 T cells during therapy. As presented in Supplement Figure S3, therapeutic effect was significantly comprised by depletion of NK or CD8 T cells, suggesting that both NK and CD8 T cells are required to achieve the cooperative benefit of the combination therapy.

### sMIC and sMIC/B10G5 complex differentially reprogram NK cell survival and proliferation

**Fig. 5:**
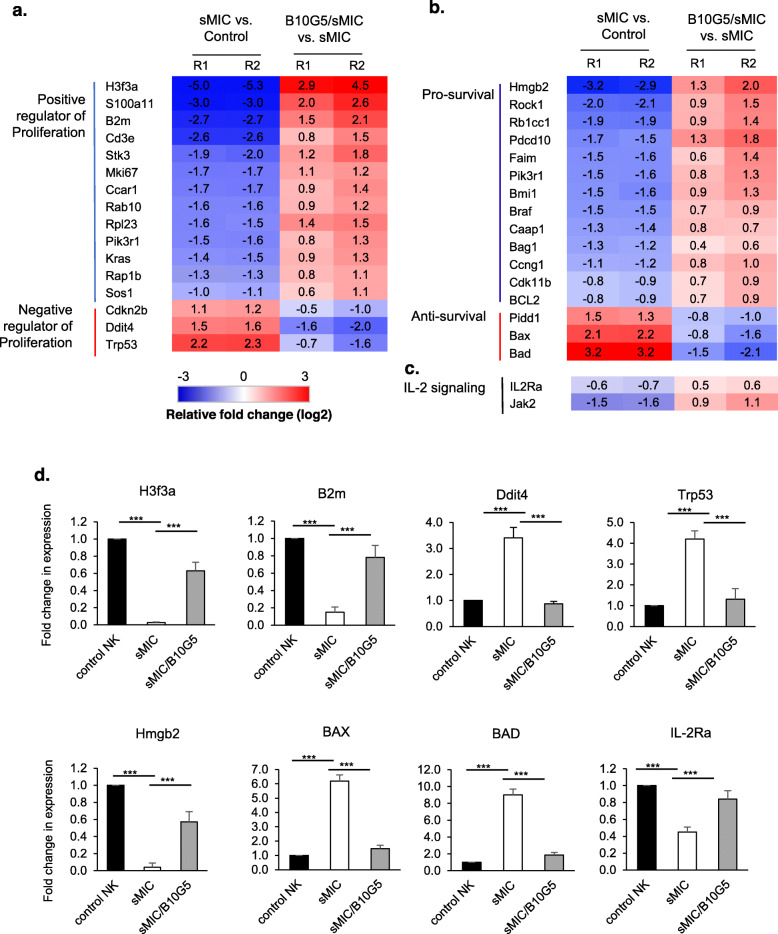
sMIC stimulation impairs the expression of genes related to NK cell homeostatic maintenance. Negatively selected mouse splenic NK cells were stimulated with recombinant MICB in the absence or presence of sMIC-clearing mAb B10G5 for 6 h. Total RNA was isolated for RNAseq analyses. R1 and R2 are replicates with two different forms of recombinant sMICB, sMICB-His tagged and sMIC-Fc tagged respectively. **a** Gene related to survival pathway that are significantly modulated in NK cells by sMIC and rescued with the sMIC-clearing mAb B10G5. **b** Gene related to proliferation pathway that is significantly modulated in NK cells by sMIC and rescued with the sMIC-clearing mAb B10G5. **c** Modification of genes in IL-2 signaling pathways by sMIC. **d** Validation of changes in the expression of representative genes as shown in **a**–**c** by qRT-PCR

One of the significant immune-suppressive effects of tumor-derived sMIC is impaired NK cell function and homeostatic maintenance [31]. B10G5 targeting sMIC has been shown to restore and enhance NK cell homeostatic maintenance [32]. We sought the underlying mechanism associated with the effect of sMIC suppressing NK cell homeostatic maintenance and how sMIC targeting can rescue NK cell maintenance. We cultured purified mouse NK cells in the presence of sMIC and sMIC plus anti-sMIC(B10G5) for 12 h and performed RNAseq and Gene Ontology (GO) analyses focusing on the survival and proliferation pathways. As shown in Fig. 5, exposure to recombinant sMIC downregulated cluster of genes positively regulating cell proliferation and survival and upregulated genes that are pro-apoptotic and inhibitors of cell cycle (Fig. 5a, b). Clearance of sMIC with the mAb B10G5 reversed the effect of sMIC (Fig. 5a, b). Notably, expression of IL-2Rα and downstream signaling molecule, such as Jak2, were also significantly upregulated with the B10G5 in the presence of sMIC (Fig. 5c). The regulation of representative genes in NK cells by sMIC and the ability to be rescued by B10G5 targeting sMIC were further confirmed by semi-quantitative RT-PCR (Fig. 5d). Collectively, these data support a potential significant mechanism whereby targeting sMIC by B10G5 antibody enhances NK cell peripheral maintenance and thus the response to PD1/PDL1 blockade therapy.

### Combination therapy of B10G5 and anti-PDL1 effectively eliminates lung metastasis

**Fig. 6:**
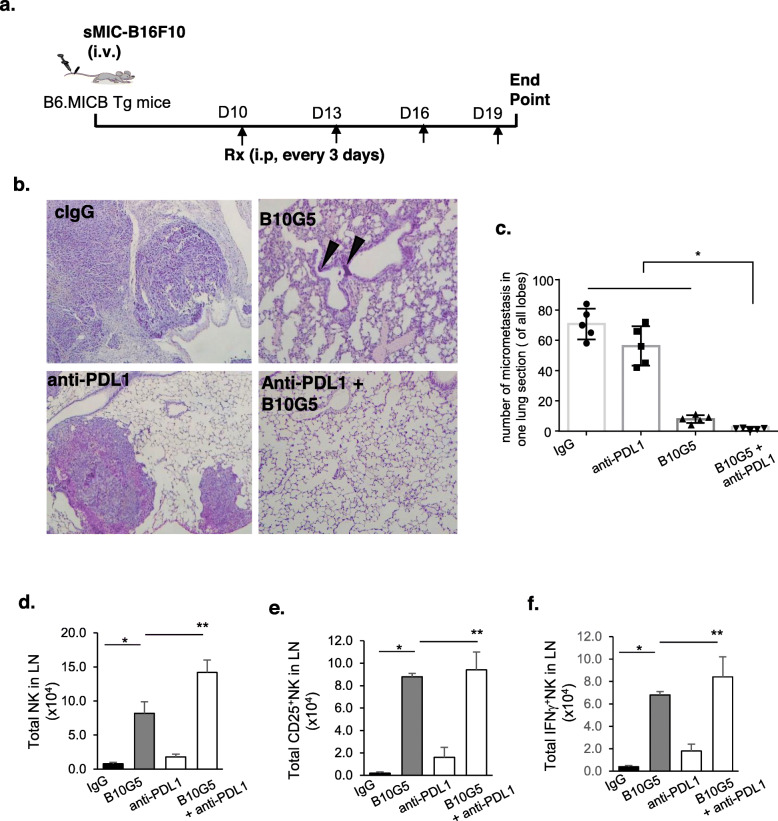
Combined therapy with B10G5 and anti-PDL1 significantly decreases established B16-sMIC^+^ metastases. **a** Depiction of treatment scheme. B6/MICB mice received i.v. injection of 4 × 10^5^ B16F10-sMICB cells. At day 10, i.p. treatment was initiated with (1) control mIgG, (2) B10G5, (3) anti-PDL1, and (4) a combination of B10G5 and anti-PDL1. Antibodies were given by i.p. injection every 3 days in 200 μl sterile PBS. **b** Representative H&E sections of lungs of mice with metastatic B16-sMICB tumors. **c** Quantitation of micrometastasis in one lung section of all lobes. **e**, **f** Enhanced NK cell numbers, CD25 expression, and IFNγ production in draining LNs (two inguinal) of animals receiving combined treatment. Data obtained at day 21 of the experiment. *p < 0.05 as compared to the control group. Arrows show micrometastasis. **p < 0.05, combination therapy as compared to monotherapy

With the proof-of-concept that combination therapy of anti-PDL1 and targeting sMIC significantly inhibits primary tumor growth as compared to single-agent therapy, we further explored the combined therapeutic effect using the experimental melanoma metastasis model. We implanted B16-sMICB tumor cells via lateral tail vein injection into syngeneic male MICB transgenic mice. At day 10 post-inoculation when pulmonary metastases were evident through random necropsy of three animals, we randomized the remaining animals into four therapeutic groups as described above with cIgG, B10G5, anti-PDL1 monotherapy, or combination therapy (Fig. 6a). Nearly all animals in the control IgG group succumbed to pulmonary metastases at day 21 post-tumor inoculation, which was designated as the study endpoint. Histology examination of the lung sections revealed that large areas of the lung from animals of the cIgG treatment group were tumors (Fig. 6b). Treatment with anti-PDL1 reduced lung micrometastasis although did not reach a statistical significance; treatment with B10G5 remarkably reduced the micrometastasis (Fig. 6b, c). Remarkably, micrometastases were rarely found in lungs from animals that received the combination therapy of anti-PDL1 mAb and B10G5 (Fig. 6b, c).

NK cells are known to be of significance in controlling tumor metastasis [5, 33, 34]. As shown in Fig. 6d–f, total number of NK cells, CD25^+^NK cells, and NK cell intrinsic responsiveness to PMA/I stimulation as presented by intracellular IFNγ staining in the draining LN was significantly increased in response to B10G5 therapy and further significantly increased with combination therapy. These data demonstrate that the reduction of lung metastasis with combination therapy is associated with significantly enhanced NK cell immunity, attributing to enhanced peripheral NK cell homeostatic maintenance and NK cell intrinsic functional potential.

### Prevalence of MIC/sMIC in metastatic melanoma lesions

**Fig. 7:**
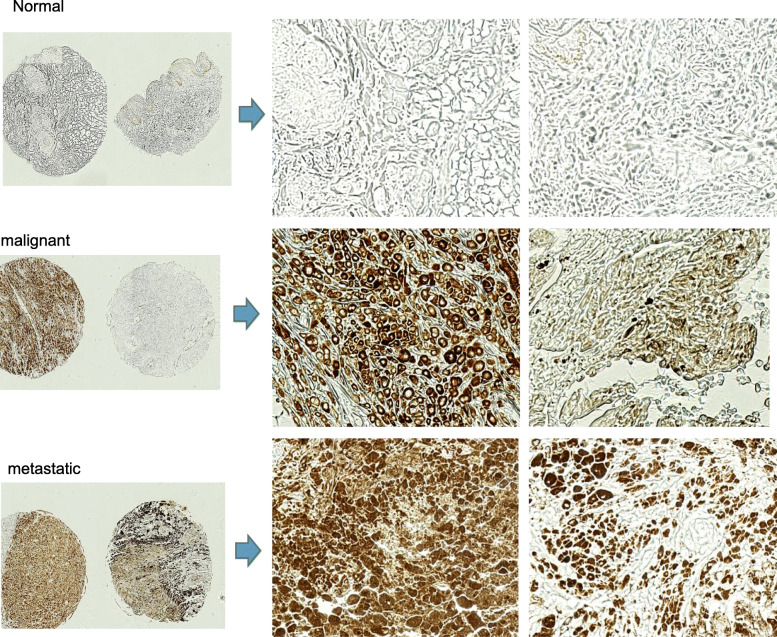
Representative MIC/sMIC abundance in metastatic melanoma tumors. The presence of MIC/sMIC was detected on TMA with a MIC (A/B)-specific antibody D4H3. Left panel, low magnification of TMA staining images. Right, higher magnification of TMA staining images

We evaluated the prevalence of MIC and sMIC in metastatic melanoma lesions using commercial source tissue microarrays (TMA), which composed of various stages of malignant melanoma tumors with different origins, lymph node (LN) metastatic lesions, benign tumors, and normal skin tissues (Supplement Figure S4a). Immunohistochemistry staining with a MIC-specific monoclonal antibody D4H3 demonstrated a high frequency of MIC/sMIC presence in melanoma tumors, with the highest frequency in LN metastatic lesions (Fig. 7 and Supplement Figure S4b). Amongst the 21 LN metastatic cases on the TMA, two cases have no evident tumors, 17 of 19 cases had tumors with strong MIC/sMIC reactivity. These data re-enforce the translation potential of the antibody B10G5 in targeting sMIC/MIC for metastatic melanoma patients.

## Discussion

With syngeneic subcutaneous and metastatic tumor models, we demonstrate in this study significantly enhanced and cooperative therapeutic effects against melanoma with the combination of the sMIC-clearing antibody B105 and PDL1/PD1 pathway blockade. We have shown that combination therapy with B10G5 and anti-PDL1 resulted in significantly more inhibition of primary tumor growth and higher degree of clearance of lung metastases than respective monotherapy. We demonstrated that the enhanced therapeutic effect with combination therapy is associated with augmented NK and CD8 T cell activation and anti-tumor potential. We also demonstrated that combination therapy cooperatively reduces expression of immunosuppressive arginase I in the tumor microenvironment and inhibits tumor angiogenesis. Interestingly, we demonstrate that expression of the receptor to enable NK cell to sensitize low dose IL-2, the IL-2R*a*, on NK cells was upregulated with B10G5 therapy and further synergistically upregulated with the combination therapy. With RNAseq analyses, we demonstrated that clearance of sMIC with B10G5 rescues the impairment of NK cell survival and proliferation pathway caused by sMIC. Finally, we demonstrate the abundance or prevalence of MIC/sMIC in metastatic melanoma tumors. Given recent clinical findings of an association between high levels of circulating NKG2D ligand sMIC and reduced response to PD1 blockade therapy in melanoma patients [4], our current study offers a viable new combination therapy to improve the response of melanoma patients to PD1/PDL1 inhibitors.

Our study demonstrates that targeting sMIC increases the IL-2 sensing receptor IL-2Rα on NK cells in vivo and that sMIC/antibody complex reprograms NK cell for homeostatic survival in vitro, although targeting sMIC enhances IL-2Ra expression on NK cells warrants further investigations. The increased IL-2Ra expression may be important for the cross-talk of NK cells and the adaptive immune response in the response to PD1/PDL1 blockade therapy. Clinical correlative studies have revealed the significance of NK cell in association with response to PD1/PDL1 blockade therapy. In metastatic melanoma patients, the expression of CD25 on NK cells has been associated with clinical response to anti-PD1 therapy [35], presumably in part due to increased NK cell sensitivity to IL-2 and thus increased survival and function. A higher density or frequency of peritumoral NK cells was found to be associated with response to anti-PD1 therapy in metastatic melanoma patients [36, 37]. A concurrent upregulation of NK cell activity related genes and MHC I in tumors that responded to PD1/PDL1 blockade therapy in melanoma patients [36]. A clustering of NK cells and stimulatory dendritic cells (DCs) was in tumors of melanoma patients who responded to anti-PD1 therapy and had prolonged survival [37]. In preclinical melanoma models, it was found that NK cells, not T cells, are required for sustaining stimulatory DC in the tumors [37]. Together, these studies underscore the significance of NK cells in mediating tumor response to PD1/PDL1 blockade therapy.

Monotherapy with B10G5 to clear sMIC was efficacious in controlling tumor growth and eliminating lung metastasis. However, combination therapy presented a cooperative and significantly enhanced effect. sMIC has been to be highly immune-suppressive via perturbing NK cell peripheral maintenance and function [19], impairing TCR/CD3 signaling by caspase-dependent destabilization of CD3ζ [28], and facilitating the expansion of MDSCs and tumor-associated macrophages [17]. These immune-suppressive effects can directly and indirectly impair CD8 T cell activation, and thus negatively impact the response to PD1 blockade therapy. We have previously shown that clearing sMIC with B10G5, a nonblocking anti-sMIC antibody, can restore NK cell homeostatic maintenance and function [18], alleviate the immune-suppressive tumor microenvironment by eliminating arginase I^+^ MDSCs and tumor-associated macrophages [17], stabilize CD3ζ expression on CD8 T cells [28], and enhance CD28-NKG2D dual co-stimulation to antigen-specific CD8 T cells [38]. These profound therapeutic effects elicited by clearing sMIC could potentiate the CD8 T cell response, in particular the tumor antigen-specific CD8 T cell response, and thus cooperatively enhance the response to PDL1/PD1 blockade therapy. Of note, the B16-sMICB tumor cell line in this study is PDL1^+^; thus, the cooperative therapeutic effect of B10G5 and anti-PDL1 demonstrated is sound. Interestingly, patients bearing tumors that are initially PDL1 negative still clinically responded to PD-1 blockade therapy [39–43]. Given that we have previously shown that clearing sMIC with B10G5 can induce the release of IFNγ [18, 28, 44], a significant regulator of PDL1 expression, one might speculate the combination of B10G5 and PD1 blockade may also be effective for tumors initially lacking PDL1 expression. Indeed, we show that clearance of sMIC rescues NK cell survival and function, presumably in part attributing to enhanced sensitivity to IL-2 released by activated antigen-specific CD8 T cells. Other studies have also presented that blocking sMIC release in preclinical models enhances NK cell function [45]. Considering that NK cells are the major IFNγ producers in active immune responses, the interaction or inter-dependence of NK cells and CD8 T cells through effector cytokine, such as IFNγ and IL-2, may account for one of the mechanisms mediating the synergistic effect of the combination therapy. Based on published studies and our current data, we propose an innate-adaptive cross-talk model that confers the cooperative therapeutic effect of targeting sMIC and PD1/PDL1 blockade (Supplement Figure S5). How NKG2D signaling and blocking PD1 signaling synergistically enhance NK cell peripheral maintenance and function as we have shown warrants a further investigation.

Checkpoint inhibitors, particularly PD1 pathway blockade, have been approved by the FDA for a number of indications, including advanced melanoma, head and neck cancer, renal cell carcinoma, non-small cell lung cancer (NSCLC), urothelial carcinoma, and metastatic Merkel-cell carcinoma, due to enhanced survival benefits compared to traditional chemotherapy. However, complete clinical responses are still limited to a small percentage of patients. The anti-PD1 antibody nivolumab only demonstrated survival benefit in advanced melanoma patients without the BRAF V600 mutation, with a 72.9% survival rate at 1 year and increase in median progression-free survival by nearly 3 months as a second-line therapy in patients that progressed after receiving ipilimumab and/or BRAF inhibitor therapy [46]. Overall, PD1/PDL1 blockade only elicited cumulative response rates of 31% in patients with melanoma, 19% in patients with NSCLC, 25% in patients with renal cell carcinoma (RCC), and 13.3% in patients with head and neck cancers [47–50]. PD1/PDL1 therapy currently is also in phase III clinical trials for the indications of BRAF V600-mutated melanoma [51], RCC [18, 30], head and neck squamous cell carcinoma (HNSCC) [31, 52], nasopharyngeal cancer [53], esophageal carcinoma [54], mesothelioma [55], hepatocellular carcinoma [48], breast cancer [20], and multiple myeloma [4]. Elevated levels of sMIC have been reported in almost all of these indications in association with reduced anti-tumor immunity or poor disease prognosis via common immune-suppressive pathways [19, 56–59]. Our current study further accentuates the potential of enhancing the efficacy of PD1/PDL1 therapy in these malignant indications by targeting sMIC.

## Conclusion

Clinical studies in melanoma patients demonstrated that the presence of soluble NKG2D ligand sMIC negatively impact the outcome of immune checkpoint blockade therapy. Our findings provide a pre-clinical proof-of-concept with novel mechanisms and translational relevance for a new avenue of antibody targeting sMIC to enhance PD1/PDL1 immune checkpoint blockade therapy for metastatic melanoma patients.

## Supplementary information

**Additional file 1.** Supplement Material and Methods. Table S1, Figures S1-S5.

## Data Availability

All data generated or analyzed during this study are included in this published article [and its supplementary information files].

## Abbreviations

MIC: MHC I chain-related molecule
PD-1: Programmed cell death protein 1
IHC: Immunohistochemistry
LN: Lymph node
dLN: Tumor-draining LN
NSCLC: Non-small cell lung cancer
TMA: Tissue microarray
