# Supplementary material for: Antibody targeting tumor-derived soluble NKG2D ligand sMIC reprograms NK cell homeostatic survival and function and enhances melanoma response to PDL1 blockade therapy
Source: J Hematol Oncol. 2020 Jun 9;13:74. doi: 10.1186/s13045-020-00896-0 (PMC7285527; doi:10.1186/s13045-020-00896-0)
Supplement: Supplementary file 1 — Additional file 1. Supplement Material and Methods. Table S1, Figures S1-S5. [file 13045_2020_896_MOESM1_ESM.pdf]

Supplement Material and Methods

Monoclonal antibody D4H3 was generated through a contracted fee-for-service vendor. Briefly, two balb/c mice were immunized with a mixture of purified recombinant soluble MICA/B (rsMICA and rsMICB) three times at two weeks intervals. A 100µg weight of the conjugated KLH peptide in Freund's complete adjuvant was used for the first immunization. At the subsequent immunizations, 50 µg of the KLH peptide in incomplete Freund's adjuvant were administered. One week prior to the last immunization, blood was collected. Antibody titers were determined by ELISA using rsMICB or rsMICA coated plates. Hybridomas were generated by fusing splenocytes from immunized mice with the murine myeloma cell line SP2/0 using polyethylene glycol. Fusion hybrids were selected using HAT medium and the reactivity of the secreted antibody was tested by ELISA and flow cytometry analysis against TRAMP, TRAMP-MICA, or TRAMP-MICB cells. Antibody produced by clone D4H3 recognizes MICA, MICB, sMICA. D4H3 was purified by BioXcell (Labnon, NH).

Table S1. Primers used for qRT-PCR

Gene	Froward	Reverse
H3f3a	GTGGTAAAGCACCCAGGAAA	ACCAGGCCTGTAACGATGAG
B2m	CCGTTCTTCAGCATTTGGAT	CTGACCGGCCTGTATGCTAT
Ddit4	CTTCTGGCTGGATGTGTATGT	AAGTGTCGAAGATCCCGAATG
Trp53	TGGAAGACTCCAGTGGGAAC	TCTTCTGTACGGCGGTCTCT
Hmgb2	GTTCCTCCCAAAGGGGATAA	TTGATCTTTGGGCGATTTTC
BAD	CCAGATCCCAGAGTTTGAGC	CAGGCCCCTATCTGTAGCAC
BAX	TGTTTGCTGATGGCAACTTC	GATCAGCTCGGGCACTTTAG
IL-2Ra	CTTCTGGCTGGATGTGTATGT	AAGTGTCGAAGATCCCGAATG
GAPDH	CTTCTGGCTGGATGTGTATGT	AAGTGTCGAAGATCCCGAATG

Supplement Figure S1

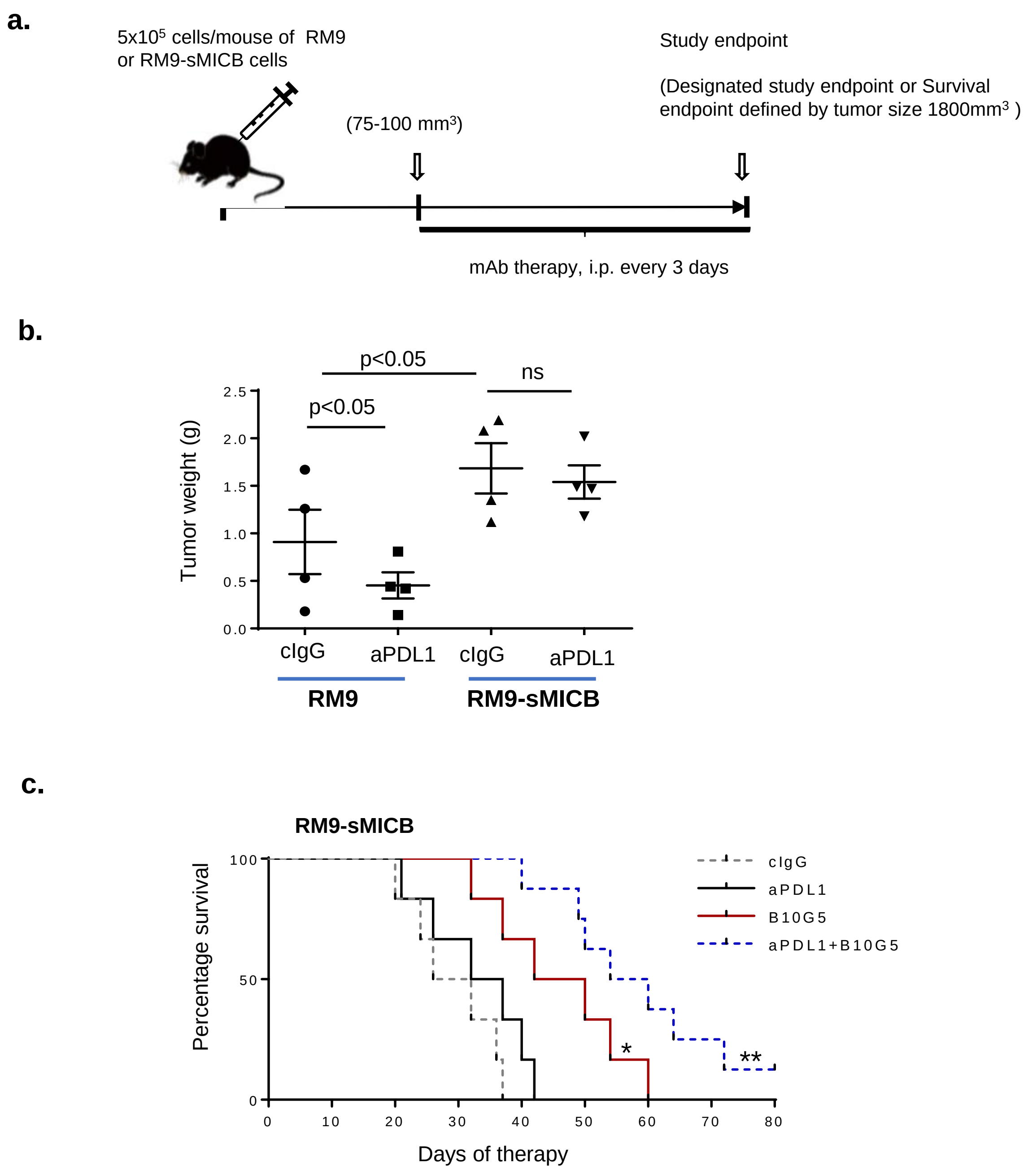

Figure S1.

Targeting sMIC with the anti-sMIC B10G5 enhances sMIC-secreting RM9 prostate tumor RM9 response to anti-PDL1 (aPDL1, clone 10F.9G2, BioXcell) **therapy. a**. Depiction of antibody therapy in the syngeneic models. $5x10^5$ RM9 or engineered sMICB-expressing RM9 cells (RM9-sMICB) cells were implanted in prostate-specific male MICB/B6 transgenic mice. When tumor volume reached 50 to 100 mm3, antibody therapy (4 mg/Kg of each respective antibody) was initiated with the dosing schedule of every 3 days till designated study endpoint as specified. **b.** Tumor weight at the specified study endpoint at which time either a designated time-frame of treatment (e.g. 6 weeks) or the 1st animal in the experiment reached survival endpoint which ever occurs first (tumor volume reaches $1800mm^3$). In this specific experiment, study endpoint is set at all animals receiving six weeks of therapy. Data show RM9-sMICB tumors are less responsive to anti-PDL1 therapy in comparison to RM9 tumors. **c.** Kaplan-Meier survival curve demonstrates that survival of mice bearing RM9-sMICB tumors was significantly improved ($p<0.05$) by B10G5 monotherapy and further significantly improved ($p<0.05$) by combination therapy of B10G5 (* $p< 0.05$) and anti-PDL1 (** $p<0.01$).

Supplement Figure S2

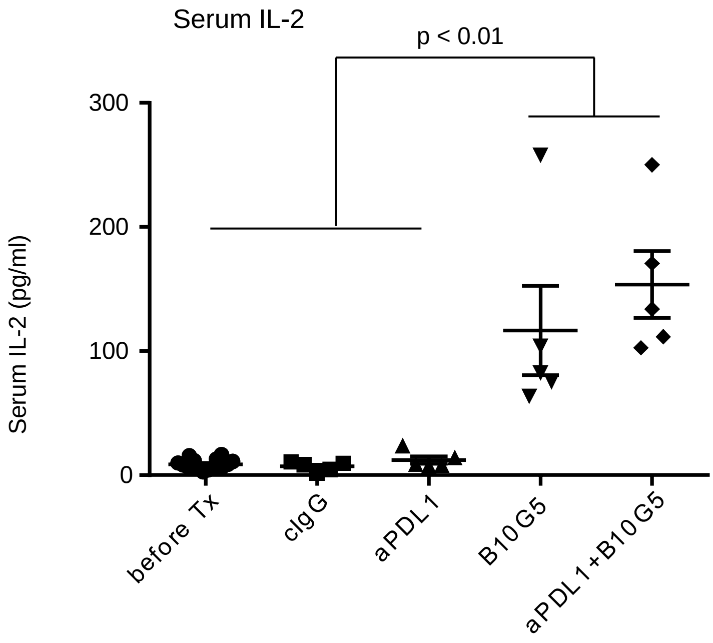

Figure S2. Serum IL-2 level before therapy (Tx) and at day 8 of respective therapy. 0.1 ml blood was collected the day before therapy (before Tx) and on day 8 of therapy via tail-veil nicking. Serum was separated. IL-2 level was measured by multiplex cytokine array assay at Eve Technologies (Calgary, Alberta, Canada). Note that there is no significant difference in serum IL-2 level among animals in any groups before therapy. Thus, the data before therapy of all animals was pooled.

Supplement Figure S3

a.

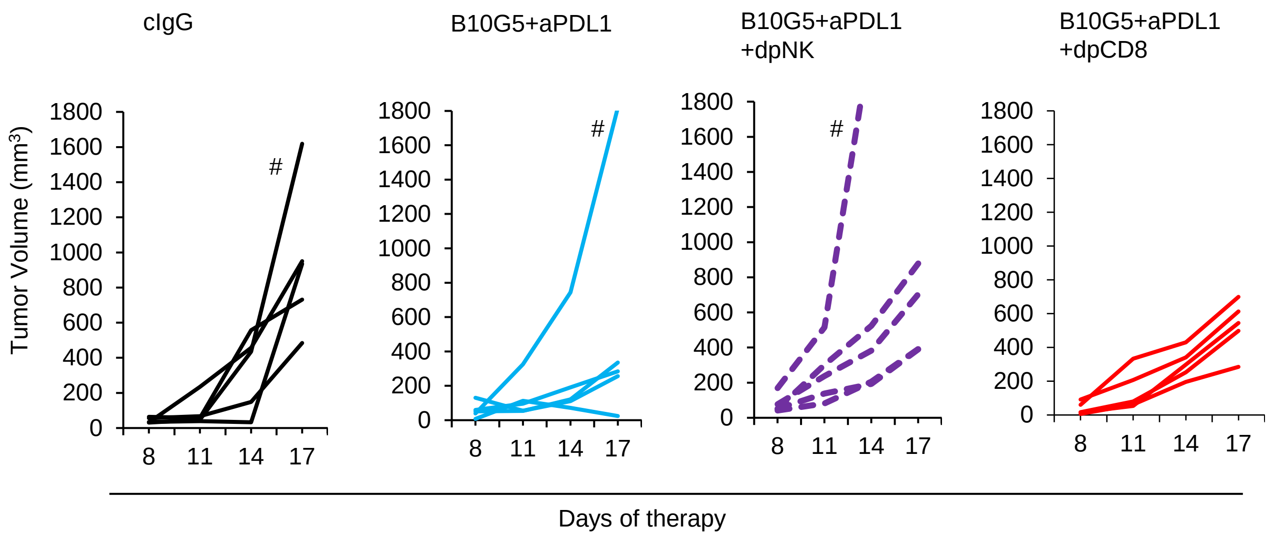

b.

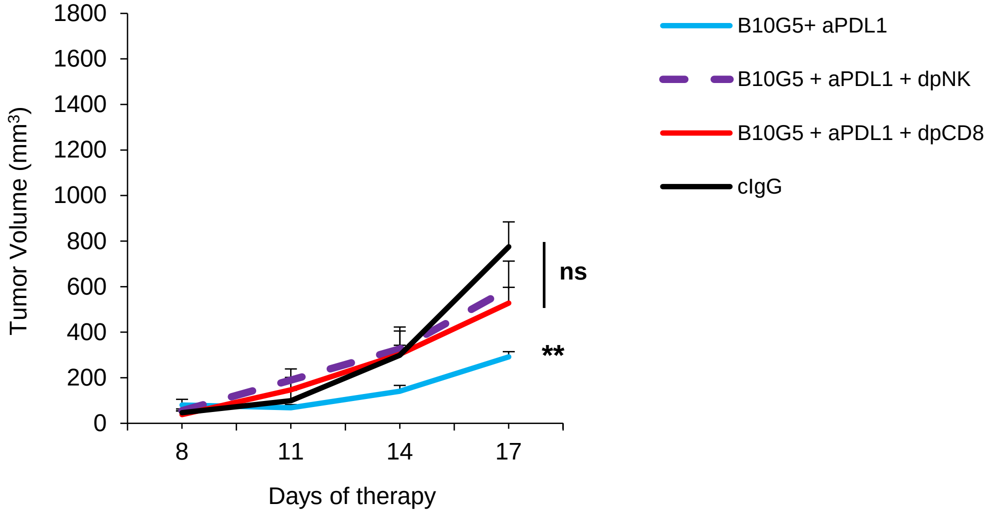

Figure S3.

Depletion of NK cells (and CD8 T cells) jeopardizes B10G5 anti-sMIC and anti-PDL1 (aPDL1) combination therapy for B16F10-sMIC tumors. B16F10-sMICB tumor cells ($4x10^5$ cells/mouse) were s.c. injected into syngeneic transgenic MICB/B6 male mice. When majority of tumors reached 75-100 mm^3 in size, animals were randomized into four groups (n=5) and receive the following treatment respectively: 1) anti-PDL1 (3 mg/Kg); 2) anti-sMIC mAb B10G5 (3 mg/KG) plus anti-PDL1 (3 mg/KG); 3) combination therapy with 6 mg/KG of ani-mouse NK1.1 (PK136, BioXcell) to deplete NK cells (dpNK); 4) combination therapy with 6 mg/Kg of anti-CD8α (clone 2.43, BioXcell) to deplete CD8 T cells (dpCD8). Therapy was given via i.p. injection every 3 days. Tumors were measured twice weekly. (**a**) Tumor growth dynamics in individual animals in each therapy group. Note that an outlier(#) of aggressive tumor growth occurred in groups of cIgG, aPDL1, and aPDL1+dpNK (**b**). Comparison of group average tumor growth (excluding the outliers in relevant groups) in therapy groups. Data show that depletion of NK cells and CD8 cells impairs tumor response to combination therapy, suggesting that both NK and CD8 T cells are important for conferring the therapeutic effect. **p<0.05 in comparison to all other groups. ns, not statistically significant.

Supplement Figure 4

S4a

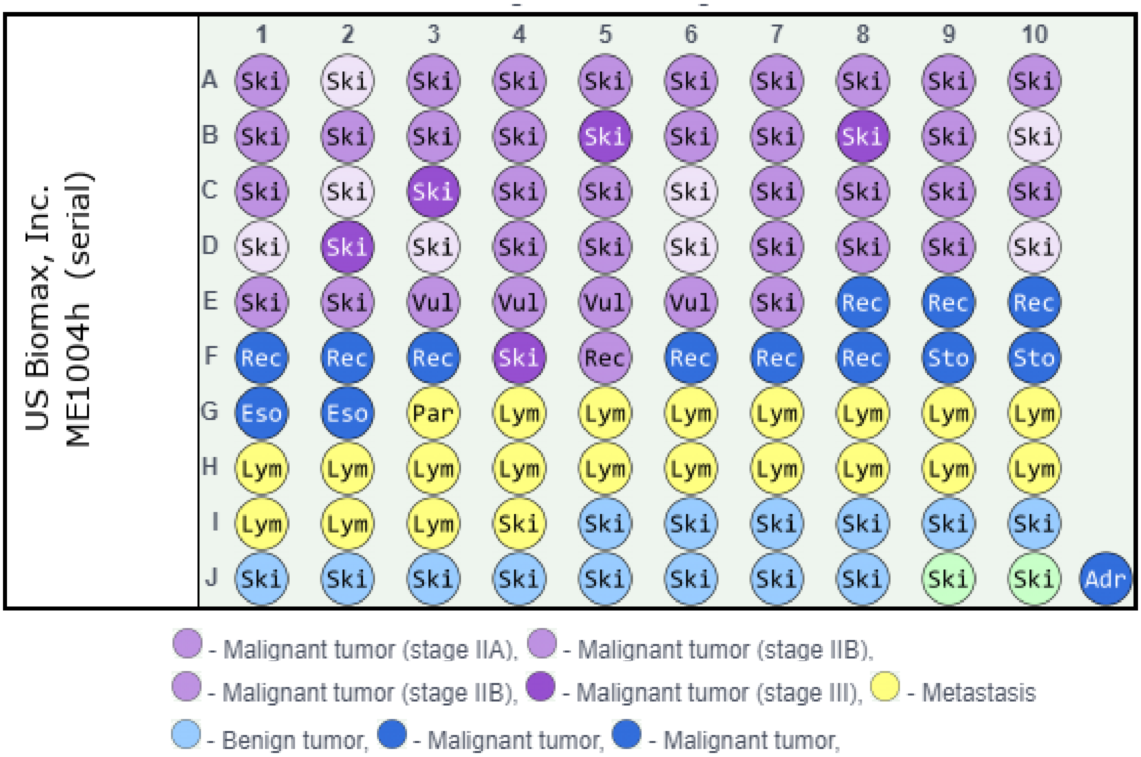

Eso, Esophagus. Rec, Rectum. Ski, Skin. Sto, stomach, Vul, Vulva. Adr, android.
Source: https://www.biomax.us/ME1004g.

S4b.

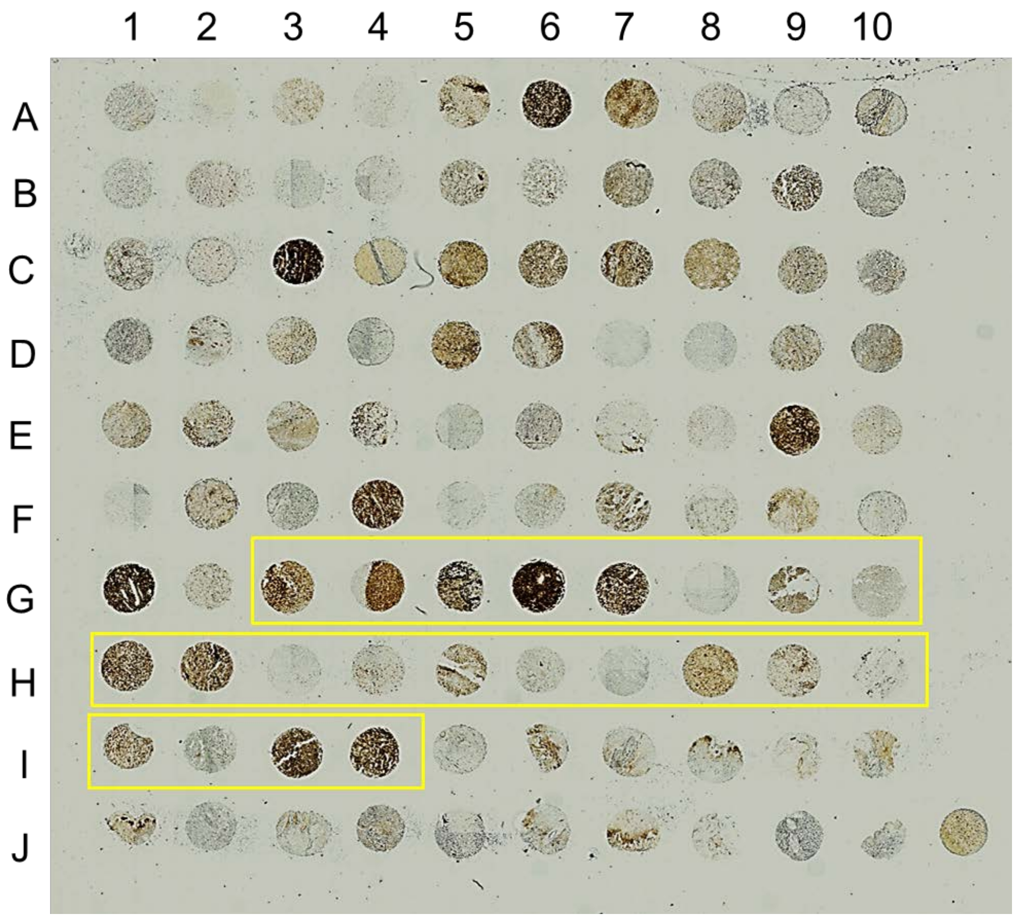

Figure S4. MIC/sMIC presence in melanoma tumors. a. Composition of the TMA array slide **. b.** IHC staining with the anti-MICA/B antibody D4H3. Boxes highlight the cases of lymph node metastasis.

Figure S5

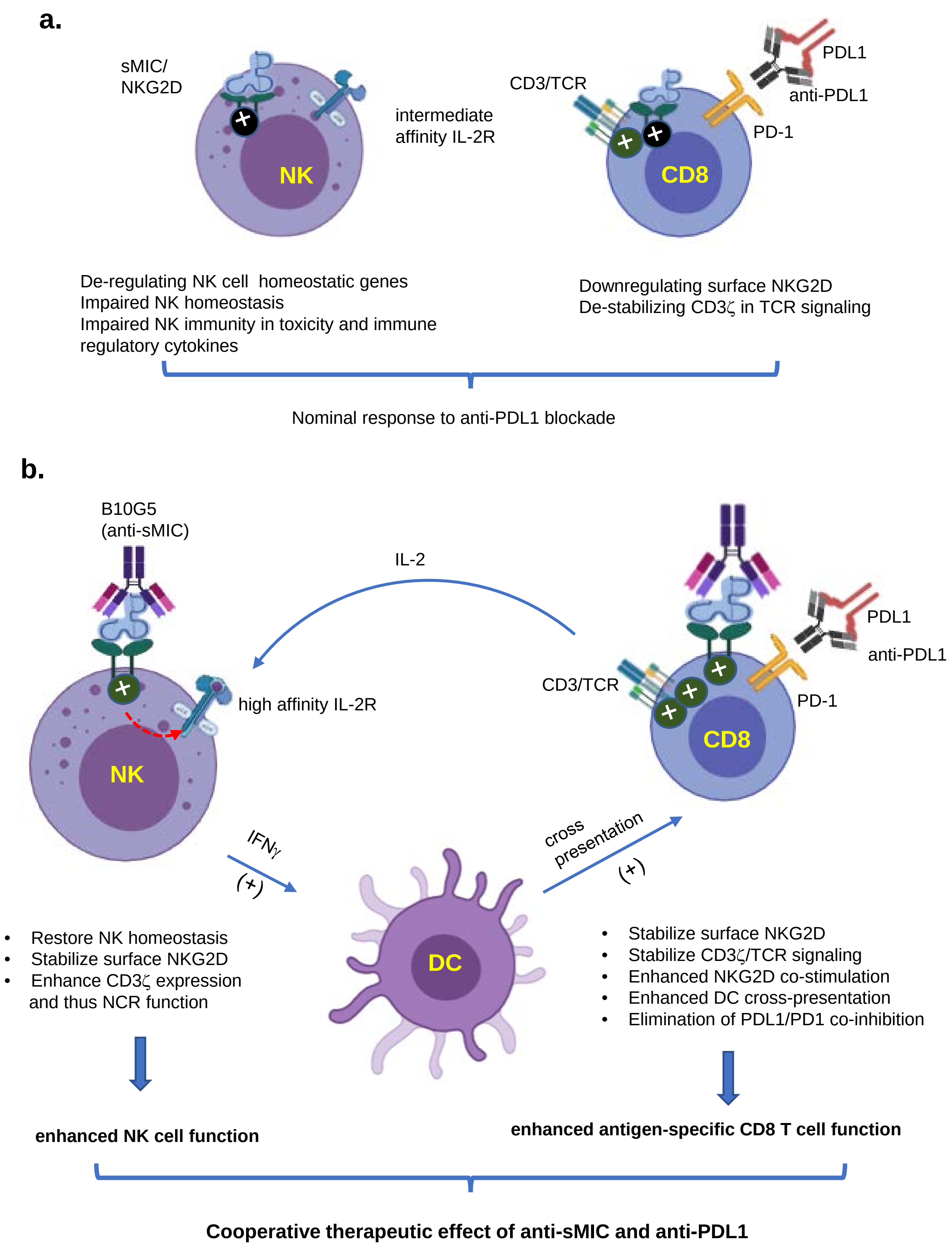
a.
sMIC/
NKG2D
NK
intermediate
affinity IL-2R
CD3/TCR
PDL1
anti-PDL1
PD-1
CD8
De-regulating NK cell homeostatic genes
Impaired NK homeostasis
Impaired NK immunity in toxicity and immune
regulatory cytokines
Downregulating surface NKG2D
De-stabilizing CD3ζ in TCR signaling
Nominal response to anti-PDL1 blockade
b.
B10G5
(anti-sMIC)
IL-2
high affinity IL-2R
NK
CD3/TCR
PDL1
anti-PDL1
PD-1
CD8
IFNγ
(+)
cross
presentation
(+)
DC
• Restore NK homeostasis
• Stabilize surface NKG2D
• Enhance CD3ζ expression
and thus NCR function
• Stabilize surface NKG2D
• Stabilize CD3ζ/TCR signaling
• Enhanced NKG2D co-stimulation
• Enhanced DC cross-presentation
• Elimination of PDL1/PD1 co-inhibition
enhanced NK cell function
enhanced antigen-specific CD8 T cell function
Cooperative therapeutic effect of anti-sMIC and anti-PDL1

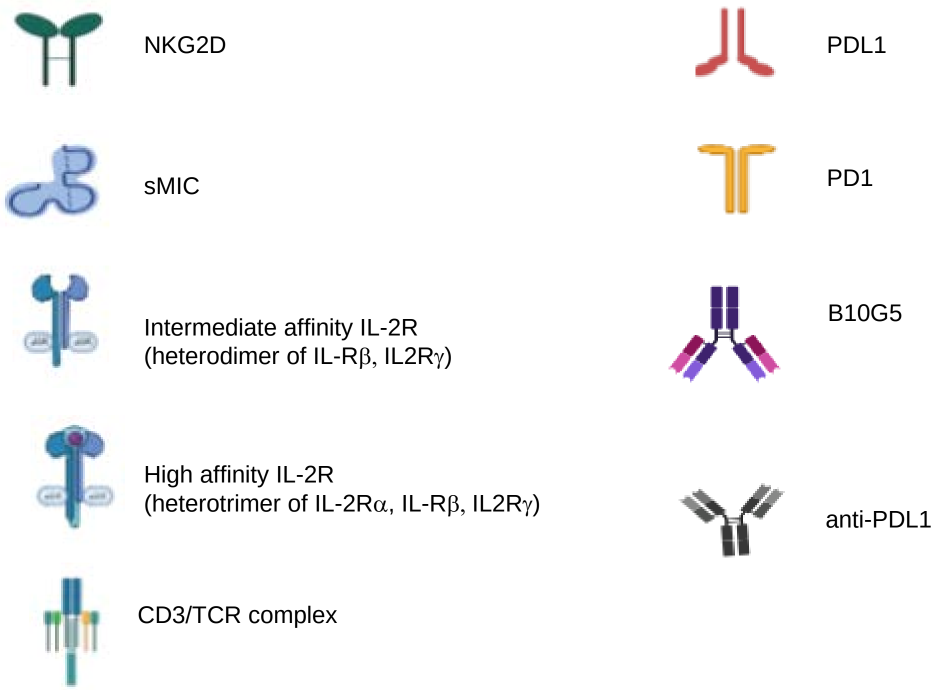

Figure S5. Proposed model of innate-adaptive cross-talk to confer the cooperative therapeutic effect of anti-PDL1 and the anti-sMIC antibody B10G5. **a**. tumor-derived sMIC has the same binding ability as cell surface membrane MIC to NKG2D and triggers NKG2D signaling intracellularly, but des-regulates genes controlling NK cell homeostatic maintenance. Intracellular sMIC/NKG2D signaling also destabilizes CD3ζ and thus impairs antigen-specific TCR signaling. Together, the effect of sMIC impairs the response to PD1/PDL1 blockade therapy. **b**. Therapy with anti-sMIC antibody B10G5 rescues the effect of sMIC on NK cell homeostatic maintenance. The complex of sMIC/B10G5 continuous to bind to NKG2D as we have shown in previous studies (Ref 38) and restores NKG2D surface expression. Complex formation by B10G5 with sMIC enhances antigen-specific CD8 T cell function through mechanisms as detailed in Ref. 38. B10G5/sMIC signals NK cells to upregulate IL-2Rα to form high affinity IL-2 receptor complex and thus allow NK cell to survive with the IL-2 secreted by activated T cells. Activated NK cells also secretes IFNγ to support DC maturation and cross-presentation of antigens to CD8 T cells. Together, the innate and adaptive cross-talk confers the cooperative therapeutic effect.
